# Evolutionary Toggling of Vpx/Vpr Specificity Results in Divergent Recognition of the Restriction Factor SAMHD1

**DOI:** 10.1371/journal.ppat.1003496

**Published:** 2013-07-18

**Authors:** Oliver I. Fregoso, Jinwoo Ahn, Chuanping Wang, Jennifer Mehrens, Jacek Skowronski, Michael Emerman

**Affiliations:** 1 Division of Human Biology, Fred Hutchinson Cancer Research Center, Seattle, Washington, United States of America; 2 Department of Structural Biology and Pittsburgh Center for HIV Protein Interactions, University of Pittsburgh School of Medicine, Pittsburgh, Pennsylvania, United States of America; 3 Department of Molecular Biology and Microbiology, Case Western Reserve School of Medicine, Cleveland, Ohio, United States of America; 4 Division of Basic Sciences, Fred Hutchinson Cancer Research Center, Seattle, Washington, United States of America; University of Massachusetts Medical School, United States of America

## Abstract

SAMHD1 is a host restriction factor that blocks the ability of lentiviruses such as HIV-1 to undergo reverse transcription in myeloid cells and resting T-cells. This restriction is alleviated by expression of the lentiviral accessory proteins Vpx and Vpr (Vpx/Vpr), which target SAMHD1 for proteasome-mediated degradation. However, the precise determinants within SAMHD1 for recognition by Vpx/Vpr remain unclear. Here we show that evolution of Vpx/Vpr in primate lentiviruses has caused the interface between SAMHD1 and Vpx/Vpr to alter during primate lentiviral evolution. Using multiple HIV-2 and SIV Vpx proteins, we show that Vpx from the HIV-2 and SIVmac lineage, but not Vpx from the SIVmnd2 and SIVrcm lineage, require the C-terminus of SAMHD1 for interaction, ubiquitylation, and degradation. On the other hand, the N-terminus of SAMHD1 governs interactions with Vpx from SIVmnd2 and SIVrcm, but has little effect on Vpx from HIV-2 and SIVmac. Furthermore, we show here that this difference in SAMHD1 recognition is evolutionarily dynamic, with the importance of the N- and C-terminus for interaction of SAMHD1 with Vpx and Vpr toggling during lentiviral evolution. We present a model to explain how the head-to-tail conformation of SAMHD1 proteins favors toggling of the interaction sites by Vpx/Vpr during this virus-host arms race. Such drastic functional divergence within a lentiviral protein highlights a novel plasticity in the evolutionary dynamics of viral antagonists for restriction factors during lentiviral adaptation to its hosts.

## Author Summary

At least 40 primate species in the wild are infected with their own lentivirus. Many of these infections arose from cross-species transmission followed by adaptation to a new host. Host antiviral proteins, called restriction factors, work to defend against both known and novel viruses and are thus engaged in a constant arms race with viral proteins. SAMHD1 is a restriction factor that blocks lentiviral infection of certain immune cells. However, SAMHD1 is counteracted by the lentiviral proteins Vpx and Vpr. Here we show that both Vpx and Vpr have evolved to recognize distinct interfaces of SAMHD1, consistent with the idea that SAMHD1 is rapidly evolving to evade this recognition. Furthermore, we show that while the site of this antagonism has been changing back and forth throughout lentiviral evolution, the mechanism by which Vpx and Vpr antagonize SAMHD1 has remained constant. These data illustrate a novel phenomenon in which there is an evolutionary toggling of divergent recognition between a restriction factor and its viral antagonist.

## Introduction

HIV-1, HIV-2, and other primate lentiviruses encode accessory virulence factors that serve to enhance viral infectivity. This is largely achieved through increasing virus replication by counteracting host antiviral proteins, known as restriction factors [1]. One such restriction factor, SAMHD1, is a deoxynucleoside triphosphate triphosphohydrolase that suppresses cellular dNTP pools [2], [3]. This prevents efficient infection of monocytes, dendritic cells (DCs), and mature macrophages by reducing the dNTP pools below the levels needed for reverse transcription of viral RNA [4], [5]. SAMHD1 has also been shown to contribute to the restriction of HIV-1 infection in resting T cells [6], [7]. SAMHD1 is composed of two structural domains: a sterile alpha motif (SAM) domain, responsible for protein-protein interactions, and a histidine-aspartic (HD) domain, responsible for the phosphohydrolase activity of the protein.

Vpx is a primate lentiviral accessory protein that antagonizes SAMHD1 function by targeting it for proteasome-mediated degradation [8], [9]. Supplying Vpx *in trans* to HIV-1 infected monocytes, DCs, macrophages, or resting T-cells alleviates the SAMHD1-mediated block of efficient reverse transcription, resulting in productive infection [6], [8], [9]. Vpx achieves this by directly binding to SAMHD1 and simultaneously to the DCAF1 substrate receptor of the CRL4 E3 ubiquitin ligase (CRL4^DCAF1^), thereby loading SAMHD1 onto this E3 complex for polyubiquitylation and subsequent degradation [8], [10]. The CRL4^DCAF1^ E3 complex usurped by Vpx consists of DCAF1, DDB1, CUL4 and RBX1 [8]–[12]. While SAMHD1 alone is unable to interact with DCAF1, Vpx bridges this interaction [8], [10], in such accelerating SAMHD1 protein turnover.

Despite its important role in lentivirus infectivity [13], Vpx is found in only two of the eight major lineages of primate lentiviruses: those of the SIVsmm/SIVmac/HIV-2 (Simian Immunodeficiency Virus of sooty mangabey, rhesus macaque, and Human Immunodeficiency Virus 2, respectively) and SIVrcm/mnd2 (Red Capped Mangabey and Mandrill viruses, respectively) lineages [14], [15]. In contrast, all extant primate lentiviruses encode for Vpr, a paralog of Vpx, which has primarily been shown to cause G2 arrest of infected cells [16]–[19]. Beyond their homology, Vpx and Vpr share functional similarities; namely they are both presumed to target host restriction factor(s) for degradation through their interaction with the CRL4^DCAF1^ complex (reviewed in [20]). Previously we showed that a subset of Vpr proteins from lentiviruses lacking Vpx are also able to degrade their host SAMHD1, and that this activity arose prior to the genesis of Vpx through recombination/duplication [21]. This indicates that the conflict between Vpx/Vpr and host SAMHD1 has been ongoing for much of primate lentiviral evolution.

One defining characteristic of restriction factors is their engagement in an evolutionary arms-race with the viruses that they inhibit [22]–[24]. This evolutionary dynamic can be identified as a signature of positive selection on host genes, such that codons accumulate mutations that result in amino acid changes at a higher frequency than expected by neutral drift. Due to the strong selective pressure imposed directly by the virus, amino acids important for the interaction between the restriction factor and its viral antagonist are often the same residues displaying the strongest signatures of positive selection [21], [24]–[26]. Indeed, SAMHD1 has undergone bursts of positive selection in the *Cercopithecinae* branch of Old World monkeys (OWM), and the amino acids in the N-terminal SAM domain of SAMHD1 that show the most significant signatures of positive selection are directly involved in the species-specific interaction of SAMHD1 with SIVmnd2 Vpx and SIVagm Vpr [21]. On the other hand, when the evolution of SAMHD1 is analyzed through a broader window of primates (including prosimians and New World monkeys) residues in the C-terminus of SAMHD1 are also found to be evolving under positive selection [27]. Furthermore, it has been shown that the C-terminus of SAMHD1 is critical for binding and degradation of SAMHD1 by Vpx from HIV-2 and SIVmac [10], [27], [28]. Thus, there is an apparent discrepancy between the sites of positive selection in SAMHD1 in OWM, where most of the primate lentiviruses exist, and the specificity of Vpx.

Here we resolve the complex specificity of Vpx/Vpr for SAMHD1 by showing that different orthologs of Vpx/Vpr have evolved multiple distinct means to target SAMHD1. While some Vpx proteins (those of HIV-2 and SIVmac) require the C-terminus of SAMHD1 for recognition, binding, ubiquitylation, and ultimately degradation of this host restriction factor, others (those of SIVmnd2 and SIVrcm) recognize the N-terminus of SAMHD1. Furthermore, by analyzing more diverse Vpr proteins, we found that this recognition is evolutionarily dynamic, with both N- and C-terminal recognition arising throughout Vpx and Vpr evolution. Thus, this viral antagonist has the capacity to evolve distinct specificities for its host target. We present a model to explain this unique phenomenon based on the head-to-tail orientation of SAMHD1 tetramers. This drastic functional divergence within a lentiviral protein highlights a novel plasticity in the evolutionary dynamics of viral antagonists for restriction factors during lentiviral cross-species transmission and adaptation to a new host.

## Results

### Different recognition of SAMHD1 by divergent Vpx proteins

**Figure 1:**
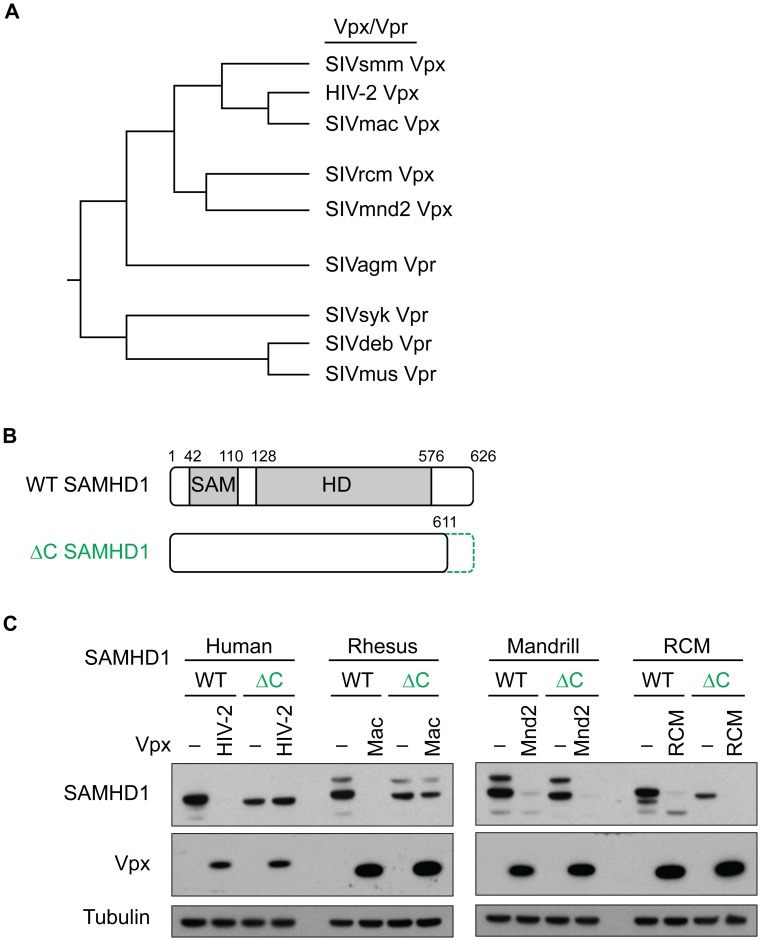
HIV-2 and SIVmac Vpx require the C-terminus of SAMHD1 for degradation. (A) Schematic Vpx/Vpr phylogenetic tree based on [21] of select lentiviral Vpx and Vpr proteins that degrade SAMHD1. (B) Schematic representation of SAMHD1 (WT) and C-terminal truncation (ΔC), including the SAM and HD domains shown in grey. The deletion at the C-terminus of SAMHD1 (green dotted line) truncates the protein at amino acid 611. Amino acid numbers are shown to indicate the relative boundaries of the domains. (C) 293T cells were transfected with HA-SAMHD1, either WT or ΔC (green), plus or minus (−) FLAG-Vpx from autologous viruses, and degradation was measured by western blotting. SAMHD1 levels are shown on the top blot, Vpx levels are shown in the middle blot, and tubulin levels are shown in the bottom blot (as a loading control). Note the differences between degradation of the WT SAMHD1 versus the ΔC SAMHD1 in the presence of some Vpx proteins, but not others.

The finding that amino acids in the N-terminus of SAMHD1 confer the specificity of SIVmnd2 Vpx and SIVagm Vpr for SAMHD1 [21], yet HIV-2 and SIVmac Vpx require the C-terminus of SAMHD1 for binding and degradation [10], [27], [28], suggested a certain complexity to the modes of SAMHD1 recognition by Vpx/Vpr. We hypothesized that different Vpx/Vpr proteins have evolved to interact with different surfaces of SAMHD1. In fact, although Vpx/Vpr proteins are related at the sequence level, phylogenetic analysis shows that there are two distinct Vpx clades that interact with SAMHD1, one containing HIV-2 and SIVmac, and another containing SIVmnd2 and SIVrcm [21] (summarized in Figure 1A). In addition, Vpr from some, but not all primate lentiviruses can degrade SAMHD1 [21] (Figure 1A). In order to assess how distinct Vpx and Vpr proteins recognize SAMHD1, we generated a set of SAMHD1 constructs, either truncated at the C-terminus or chimeric at the N-terminus, and assayed for the ability of multiple Vpx/Vpr proteins to degrade these SAMHD1 proteins. Chimeras and truncations were based on previous results which showed that SAMHD1 is still catalytically active and able to form tetramers when truncated at the C-terminus or the N-terminus [29].

First, we looked at the dependence of Vpx on the C-terminus of SAMHD1. Previous results have shown that truncation of the last 15 amino acids of human SAMHD1 abrogated the interaction of SIVmac and HIV-2 Vpx with SAMHD1 [10], [27]. To test for degradation of a C-terminally truncated version of SAMHD1 (ΔC SAMHD1, truncated from amino acid 612–626) (Figure 1B), we co-transfected human 293T cells with FLAG-tagged Vpx and HA-tagged SAMHD1, and assayed for a decrease of SAMHD1 expression. We initially tested HIV-2 Vpx, SIVmac Vpx, SIVmnd2 Vpx and SIVrcm Vpx for their ability to degrade their own host species SAMHD1. As expected, each Vpx tested was able to degrade its autologous host SAMHD1 (Figure 1C). However, while HIV-2 and SIVmac Vpx were unable to degrade SAMHD1 truncated at the C-terminus (Figure 1C, left panel), neither SIVmnd2 nor SIVrcm Vpx required the C-terminus of SAMHD1 for degradation, as both Vpx proteins were readily able to degrade WT and ΔC constructs (Figure 1C, right panel). This suggests that these Vpx proteins evolved differences in their specificity determinants for degradation of SAMHD1, possibly due to the selective pressure on SAMHD1. We also assayed a panel of phylogenetically distinct HIV-2 Vpx genes representing clades A, B, and H as well as Vpx from SIVsmm (Figure S1A) for degradation of WT and ΔC human SAMHD1. We found that while all but one of the Vpx tested could degrade full-length SAMHD1, none was able to degrade the ΔC construct (Figure S1B). This suggests that the dependence on the C-terminus of SAMHD1 is indeed conserved within the diversity of HIV-2 Vpx and its precursor SIVsmm Vpx, but is different from the requirements of SIVmnd2 and SIVrcm Vpx to degrade SAMHD1.

**Figure 2:**
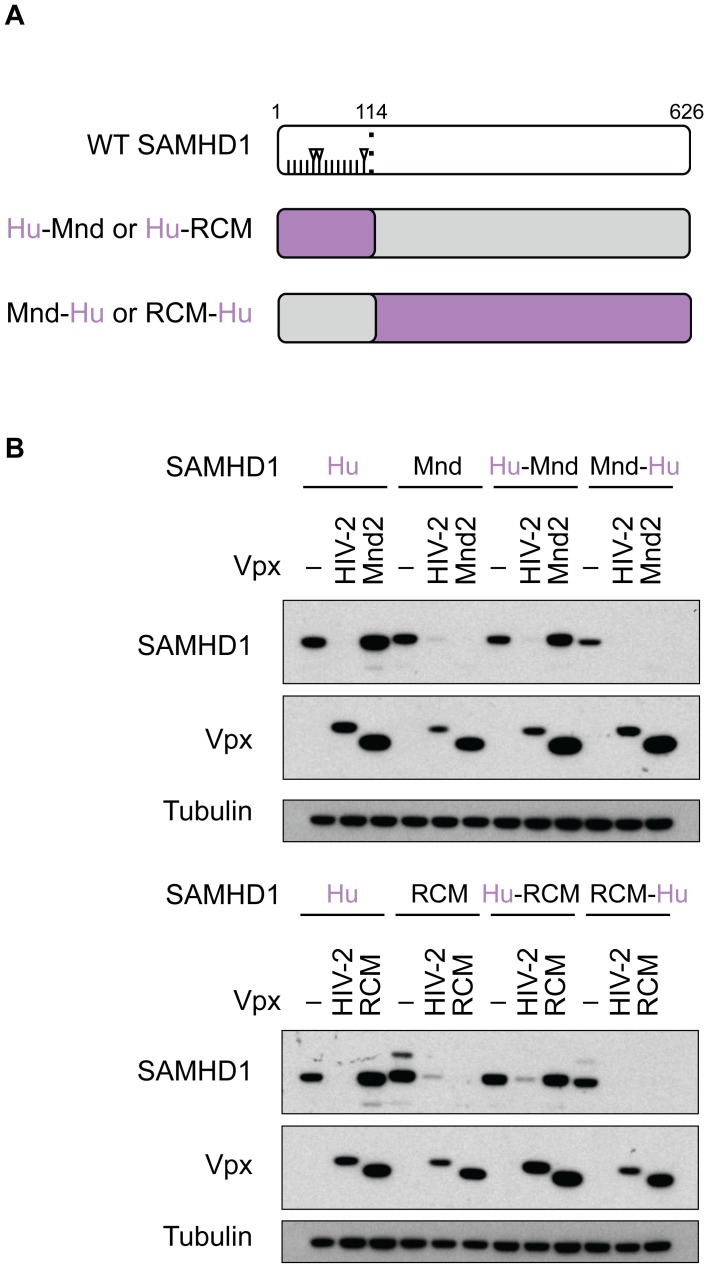
SIVmnd2 and SIVrcm Vpx require the N-terminus of SAMHD1 for degradation. (A) Schematic representation of WT SAMHD1 and chimeric proteins. Amino acids in the N-terminus that differ between human (Hu) SAMHD1 and mandrill (Mnd) or RCM SAMHD1 are shown as tick-marks, while codons that differ and have been shown to be evolving under strong positive selection (amino acids 32, 36, and 107) are shown with arrowheads. The regions of the chimeric SAMHD1 protein coming from the human gene are shown in purple, while the regions coming from mandrill or RCM SAMHD1 are shown in grey. (B) 293T cells were transfected with HA-tagged WT or chimeric SAMHD1, plus or minus (−) FLAG-Vpx, and degradation was measured by western blotting as described in the legend for Figure 1.

Because we have previously shown that amino acids under positive selection in the N-terminus of SAMHD1 affect binding and degradation by SIVmnd2 Vpx [21], we next tested the effects of natural variation in the N-terminus of SAMHD1 on Vpx-mediated degradation by diverse Vpx proteins. We therefore generated SAMHD1 chimeras by swapping the first 114 amino acids (including the N-terminus and the SAM domain) of human SAMHD1 with those of either mandrill or RCM SAMHD1 (Figure 2A). Human SAMHD1 was used for these chimeras because it is quite divergent at the N-terminus with 12 or 13 amino acids that differ relative to mandrill or RCM, respectively, three of which show strong signatures of positive selection (Figure 2A and Figure S2). Notably, neither SIVmnd2 Vpx (Figure 2B, top panels) nor SIVrcm Vpx (Figure 2B, lower panels) were able to degrade human SAMHD1, while HIV-2 Vpx was able to degrade both mandrill and RCM SAMHD1. Importantly, when we replaced the first 114 residues of mandrill or RCM SAMHD1 with that of human, neither SIVmnd2 nor SIVrcm Vpx were able to degrade these chimeric SAMHD1 proteins. However, both SIVmnd2 Vpx and SIVrcm Vpx (as well as HIV-2 Vpx) were able to degrade the respective reciprocal SAMHD1 chimeras, where either mandrill or RCM N-terminus was fused with the rest of human SAMHD1 (Figure 2B). Thus, the N-terminus of mandrill and RCM SAMHD1 are sufficient to confer degradation of human SAMHD1 by SIVmnd2 and SIVrcm Vpx, respectively. These results demonstrate that while SIVmnd2 and SIVrcm are insensitive to truncations at the C-terminus of SAMHD1 (Figure 1C), the species-specific sequence differences within the N-terminus of SAMHD1 are necessary and sufficient for degradation by SIVmnd2 and SIVrcm Vpx. On the other hand, HIV-2 Vpx (and SIVmac Vpx, data not shown), which depends on the C-terminus of SAMHD1 for degradation (Figure 1C), is not affected by the natural sequence variation present in the N-terminus of mandrill or RCM SAMHD1 (Figure 2).

**Figure 3:**
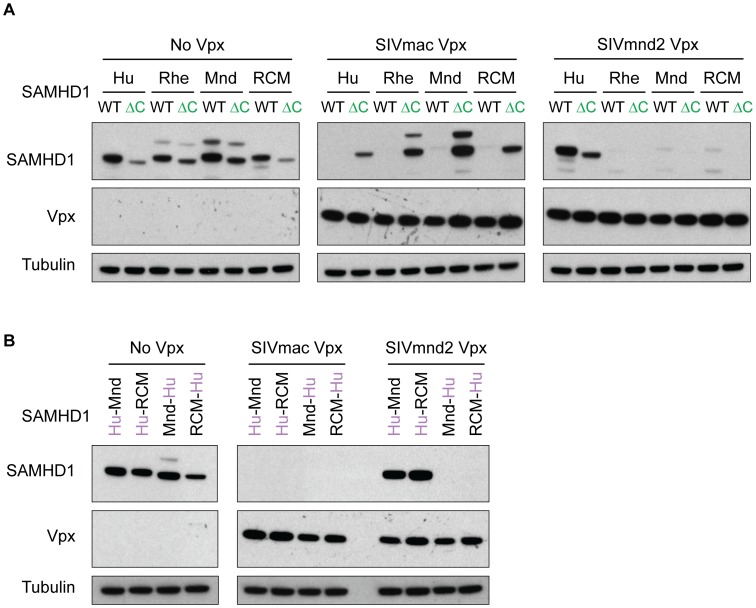
Vpx specificity for N- versus C-terminal binding of SAMHD1 is independent of the SAMHD1 homologue. (A) Empty vector control, C-terminal binding FLAG-SIVmac Vpx, or N-terminal binding FLAG-SIVmnd2 Vpx were co-transfected in 293T cells with HA-SAMHD1, either WT or ΔC, from multiple primate species, and degradation of SAMHD1 was measured by western blotting. (B) Same as in (A) except chimeric SAMHD1 proteins were used instead of WT or ΔC.

We also wanted to determine if a Vpx shows the same requirement for either the C-terminus or changes in the N-terminus regardless of the SAMHD1 it is challenged with. Therefore, we assayed for the ability of the divergent SIVmac or SIVmnd2 Vpx to degrade multiple species' SAMHD1, either C-terminal deleted or chimeric at the N-terminus (Figure 3). Similar to the results with the autologous SAMHD1, we found that SIVmnd2 Vpx degrades both wild type and ΔC SAMHD1 from rhesus, mandrill, and RCM, while SIVmac was unable to degrade ΔC SAMHD1 from any of the four species, though it was able to degrade the wild type protein from all (Figure 3A). Conversely, when SIVmac Vpx was assayed against chimeric SAMHD1 constructs (Figure 3B), it was able to degrade all four constructs, regardless of the sequence at the N-terminus. However, SIVmnd2 Vpx was unable to degrade those with human N-termini (Figure 3B). Together, these data indicate that Vpx from distinct viral lineages have evolved different requirements for degradation of SAMHD1 and that these requirements are intrinsic to Vpx.

### Vpx degrades SAMHD1 through a conserved mechanism, despite N- or C-terminal binding

**Figure 4:**
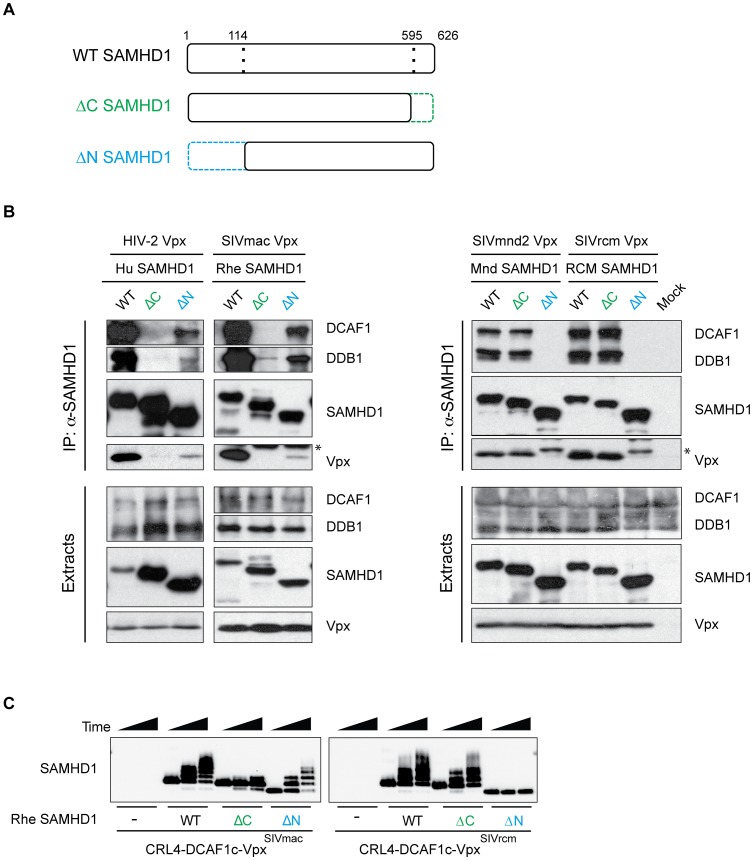
N- and C-terminal binding Vpx proteins degrade SAMHD1 through a conserved mechanism. (A) Schematic representation of wild type (WT) SAMHD1, C-terminally truncated SAMHD1 (ΔC, shown in green), and N-terminally truncated SAMHD1 (ΔN, shown in blue). Amino acid numbers of the truncations are shown, with dotted line indicating the truncated region of SAMHD1. (B) HA-SAMHD1 (WT, ΔC, and ΔN) were transiently co-expressed in 293T cells with the autologous FLAG-Vpx and immunoprecipitated from whole cell extracts with anti-HA resin. HA-SAMHD1, FLAG-Vpx, DCAF, and DDB1 were detected in immune complexes (top panels) or extracts (bottom panels) by western blotting. * denotes the antibody light-chain. (C) *In vitro* ubiquitylation of WT, ΔC, and ΔN rhesus SAMHD1, in the presence or absence of SIVmac Vpx (left panel) or SIVrcm Vpx (right panel). SAMHD1 and Vpx were incubated with Cul4, DCAF1c, RBX1, UBA1 (an E1 enzyme), UbcH5b (an E2 enzyme) and FLAG-tagged ubiquitin for increasing time (0, 15, 30 min), and ubiquitylation of each SAMHD1 construct was analyzed by western blotting.

SIVmac Vpx has been shown to target human SAMHD1 for proteasome-mediated degradation by interacting directly with SAMHD1 as well as the E3 ubiquitin ligase complex consisting of DCAF1, DDB1, and CUL4-RBX1 [10]. However, with such disparate requirements for SAMHD1 degradation between SIVmac and SIVmnd2/SIVrcm Vpx, we asked if the N-terminal binding SIVmnd2 and SIVrcm Vpx proteins utilize the same DCAF1 substrate receptor and E3 complex as the C-terminal binding SIVmac Vpx. We assayed for interaction of SAMHD1 with Vpx and endogenous DCAF1 and DDB1 via co-immunoprecipitations (co-IP) in 293T cells (Figure 4). The presence of DCAF1 and DDB1 in the co-IP represents formation of a CRL4^DCAF1^ complex that includes SAMHD1 and Vpx. We found that, similar to human and rhesus SAMHD1, full-length mandrill and RCM SAMHD1 interact with Vpx from their autologous virus, as well as the ubiquitin ligase components DCAF1 and DDB1 (Figure 4B, WT SAMHD1 lanes). Consistent with previous results [10], we found that SAMHD1 does not interact with DDB1 and/or DCAF1 in the absence of Vpx (Figure S3B and S3C). Thus, formation of the CRL4^DCAF1^ complex is conserved between Vpx proteins, regardless of whether the Vpx protein utilizes the N- or C-terminus of SAMHD1 for binding.

These results allowed us to next ask whether the failure to degrade either N- or C-terminally truncated SAMHD1 is due to the inability of Vpx proteins to recruit the mutant SAMHD1 proteins to the E3 complex. We performed co-IP analyses of both C-terminally truncated (ΔC) and N-terminally truncated (ΔN) SAMHD1 (Figure 4A) in 293T cells, and assayed for interaction with Vpx, DCAF1 and DDB1 (Figure 4B). In agreement with the degradation profiles, both human and rhesus ΔC SAMHD1 were unable to bind to their autologous Vpx and consequently to DDB1 and DCAF1 (Figure 4B, left panel), while the ΔN truncations show a less dramatic effect on binding. Conversely, truncating mandrill or RCM SAMHD1 at the C-terminus had no effect on Vpx, DDB1, or DCAF interaction, yet ΔN SAMHD1 truncations completely blocked these interactions. These data were further confirmed through gel filtration size exclusion chromatography of SAMHD1-Vpx-DDB1-DCAF1c complexes formed with purified recombinant proteins *in vitro* (Figure S3), such that SIVmac Vpx was able to recruit WT and ΔN SAMHD1 to a DDB1-DCAF1c complex, but not ΔC SAMHD1 (Figure S3D; controls without Vpx-DDB1-DCAF1c, without SAMHD1, and without Vpx in Figure S3A, B, and C, respectively). On the other hand, SIVrcm Vpx was unable to recruit a ΔN SAMHD1 from rhesus, yet it was readily able to interact with WT and ΔC rhesus SAMHD1 (Figure S3E). In agreement with the degradation data, these data suggest that the inability of Vpx to degrade SAMHD1 is due to an inability to recruit SAMHD1 to the DCAF1 subunit of the E3 complex. Furthermore, regardless of the precise requirement for SAMHD1 binding, all Vpx tested can utilize the same CRL4^DCAF1^ E3 ubiquitin ligase to antagonize SAMHD1.

Finally, to show that these interactions result in the ubiquitin-mediated degradation of SAMHD1, we performed an *in vitro* ubiquitylation assay [10]. We found that when WT, ΔC, or ΔN rhesus SAMHD1 was incubated with a preformed Cul4-DCAF1c-Vpx^SIVmac^ E3 ubiquitin ligase complex, WT and ΔN SAMHD1 were readily ubiquitylated in the presence of Vpx, however ΔC SAMHD1 was not (Figure 4C, left panel). Conversely, when these SAMHD1 proteins were incubated with CRL4-DCAF1c-Vpx^SIVrcm^, WT and ΔC SAMHD1 were ubiquitylated, however ΔN SAMHD1 was not (Figure 4C, right panel). This is in agreement with our previous data and demonstrates a conserved pathway of ubiquitin-mediated degradation despite different N- and C-terminal SAMHD1 binding by different Vpx proteins.

### SIVrcm Vpx utilizes a distinct interface to recognize SAMHD1

Our data showing that all Vpx-SAMHD1 complexes bind DCAF1 and the DDB1 module of CRL4^DCAF1^ suggests that the region of Vpx that binds this E3 ubiquitin ligase complex has been conserved throughout lentiviral evolution. However, our observation that different Vpx proteins bind distinct regions of SAMHD1 raised the possibility that the Vpx surface utilized to bind SAMHD1 has changed during primate lentiviral evolution. Thus, we wanted to investigate if N-terminal binding Vpx proteins require the same residues in Vpx to degrade SAMHD1 as C-terminal binding Vpx proteins.

**Figure 5:**
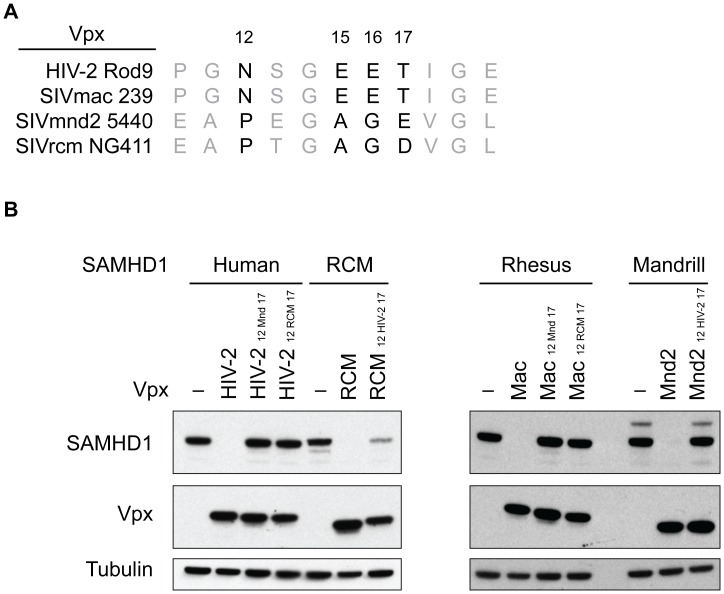
SIVrcm Vpx utilizes a unique interface to degrade SAMHD1. (A) Alignment of the amino acids in Vpx that have been previously shown to be important for SIVmac Vpx-mediated degradation of SAMHD1, highlighted in in black with amino acid numbers above. (B) Wild type and mutant Vpx constructs were tested for their ability to degrade SAMHD1 by cotransfection in 293T cells, and analyzed by western blotting. HIV-2_12 Mnd 17_ and Mac_12 Mnd 17_ (or HIV-2_12 RCM 17_ and Mac_12 RCM 17_) indicates the amino acids 12 through 17 of HIV-2 Vpx and SIVmac Vpx that have been changed to the corresponding amino acids found in SIVmnd2 Vpx (or SIVrcm Vpx), while RCM_12 HIV-2 17_ and Mnd2_12 HIV-2 17_ indicates that amino acids 12 through 17 of SIVrcm and SIVmnd2 Vpx have been changed to the corresponding amino acids found in HIV-2/SIVmac Vpx. – indicates no Vpx empty vector control.

It has been previously shown that amino acids 12, 15, 16, and 17 are necessary for the ability of C-terminally binding SIVmac Vpx to interact with human SAMHD1 in order to load it onto DCAF1 for degradation [10] and to subsequently allow for viral infectivity in macrophage and dendritic cells [30]. However, the amino acids at positions 12, 15, 16, and 17 in SIVmnd2 and SIVrcm Vpx differ from those of HIV-2 and SIVmac Vpx (Figure 5A), suggesting the possibility that differences in this region may be important for N- and C-terminal recognition. Therefore, we attempted to change the specificity of these Vpx proteins for the N- or the C-terminus by swapping amino acids 12/15/16/17 in SIVmnd2 and SIVrcm to those of HIV-2 Vpx and SIVmac Vpx, as well as the reciprocal change of HIV-2 Vpx and SIVmac Vpx to those of SIVmnd2 and SIVrcm. Changing these residues in both HIV-2 and SIVmac Vpx abolished the activity of these proteins against their host SAMHD1 (Figure 5B), indicating that the precise residues are necessary for HIV-2 and SIVmac Vpx to degrade SAMHD1. These changes also rendered SIVmnd2 Vpx unable to degrade mandrill SAMHD1 (Figure 5B), suggesting that though the specific residues differ between HIV-2/SIVmac and SIVmnd2 Vpx, the precise residues at this position are also required for recognition of SAMHD1 by SIVmnd2 Vpx. Interestingly, a SIVrcm Vpx mutant at these positions was still able to degrade RCM SAMHD1, although with slightly decreased activity (Figure 5B). Importantly, these amino acid changes did not change the specificity of SIVrcm Vpx for the N-terminus of SAMHD1 versus the C-terminus of SAMHD1 (Figure S4). Nonetheless, these data suggest that SIVrcm Vpx utilizes a distinct interface to bind to RCM SAMHD1 for recruitment to the CRL4^DCAF1^ E3 complex.

### Evolutionary “toggling” of Vpx/Vpr specificity towards SAMHD1

**Figure 6:**
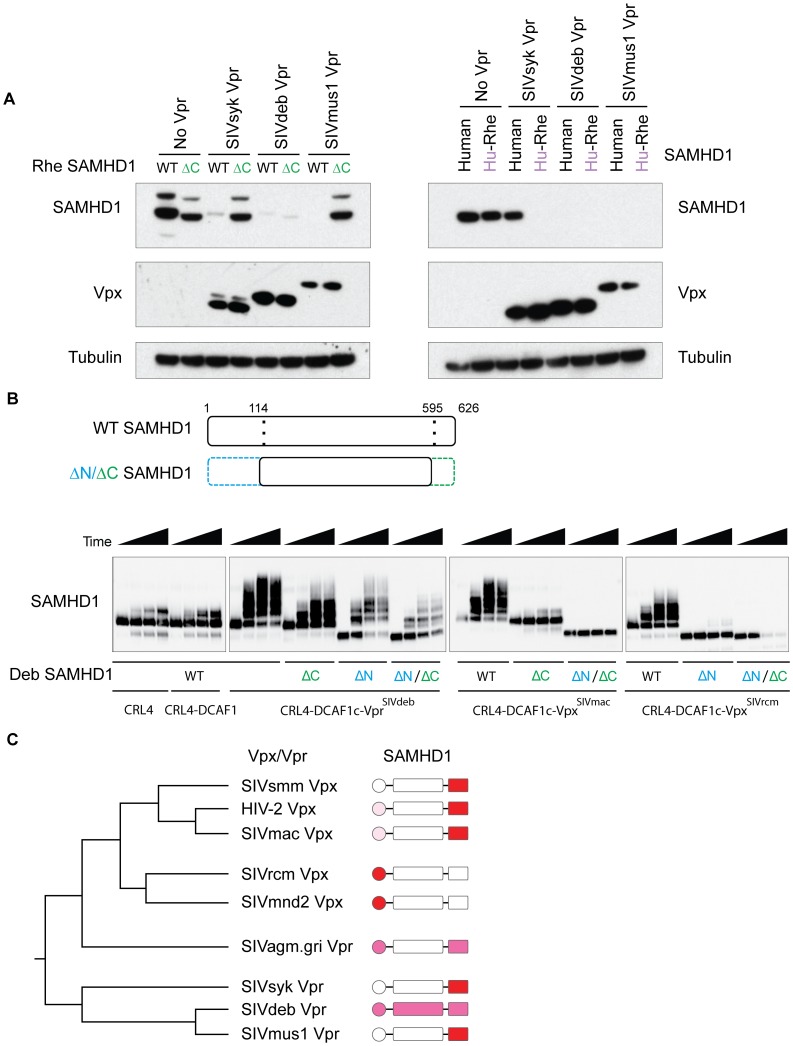
Vpx and Vpr have toggled throughout evolution in their requirement for the N- or the C-terminus of SAMHD1. (A) 293T cells were transfected with HA-SAMHD1, either WT or ΔC (left panel), or WT or chimeric Human-Rhesus SAMHD1 (consisting of residues 1–114 of human SAMHD1 and 115–626 of rhesus SAMHD1, right panels), plus or minus FLAG-Vpr, and degradation was measured by western blotting as described in the legend for Figure 1. Rhesus SAMHD1 was used as all Vpr tested herein can degrade this SAMHD1. Human SAMHD1 was used in N-terminal chimeras as it contains multiple non-synonymous changes compared to other primate SAMHD1. (B) *In vitro* ubiquitylation of WT, ΔC, ΔN, and ΔN/ΔC DeBrazza's SAMHD1 (consisting of amino acids 115–595, schematic representation in top panels), in the presence or absence of SIVdeb Vpr, SIVmac Vpx, or SIVrcm Vpx. CRL4 and CRL4-DCAF1 alone were used as controls. Experiment preformed as in Figure 4B and ubiquitylation of each SAMHD1 construct was analyzed by western blotting. Timepoints shown are 0, 15, 30, and 60 minute incubations. (C) Schematic Vpx/Vpr phylogenetic tree as in Figure 1A and SAMHD1 diagrams depicting dependence of Vpx/Vpr on the N- or C-terminus of its autologous SAMHD1. Red indicates strong dependence based on all assays. Light pink indicates slight dependence based on co-IP data. Magenta indicates intermediate dependence.

Our previously published data suggests that the ability of Vpx to degrade SAMHD1 arose first in Vpr, prior to the duplication/recombination event that led to the genesis and subsequent subfunctionalization of Vpx [21]. Given our results that SAMHD1 recognition is distinct between different Vpx lineages, we wished to determine how the specificity for SAMHD1 evolved in Vpr proteins that arose prior to the Vpx duplication (e.g. SIVdeb, SIVsyk, and SIVmus Vpr, Figure 1A). We found that SIVsyk and SIVmus Vpr, like HIV-2 and SIVmac Vpx, were unable to degrade a rhesus SAMHD1 that was truncated at the C-terminus (Figure 6A, left panels), suggesting a C-terminal dependence. We further assayed for the dependence on natural variation at the N-terminus of SAMHD1 by testing each Vpr for their ability to degrade human SAMHD1 and a chimeric rhesus SAMHD1 with human SAMHD1 N-terminus (schematic as in Figure 2A). We found that SIVsyk Vpr cannot degrade human SAMHD1, but can degrade the rhesus SAMHD1 chimera with a human N-terminus (Figure 6A, right panels). This indicates that determinants for SIVsyk Vpr are not at the N-terminus of SAMHD1, and is consistent with the inability of SIVsyk Vpr to degrade C-terminally truncated SAMHD1 (Figure 6A, left panels). SIVmusVpr is able to degrade both human SAMHD1 and the human-rhesus chimeric SAMHD1, which further suggests that SIVmus Vpr is not affected by natural variation in the N-terminus (Figure 6A, right panels). These results suggest that both SIVmus and SIVsyk Vpr depend primarily on the C-terminus of SAMHD1 for degradation.

SIVdeb Vpr is interesting because its ability to degrade SAMHD1 is not affected by truncation of the C-terminus of SAMHD1 (Figure 6A, left panels), yet it is also not affected by natural variation of human SAMHD1 at the N-terminus either (Figure 6A, right panels). This result is consistent with the previous finding that SIVdeb Vpr can degrade SAMHD1 from all primate species tested [21], and suggests that determinants at the N- and C-terminus are partially redundant for recognition by SIVdeb Vpr. SIVagm.gGri Vpr also appears to involve interactions at both N- and C- termini (Figure S5).

To directly test the specificity of SIVdeb Vpr for SAMHD1, we used purified recombinant proteins to assay for the ability of SIVdeb Vpr to *in vitro* ubiquitylate DeBrazza's SAMHD1 lacking the N-terminus, the C-terminus, or both the N- and C-termini (ΔN/ΔC SAMHD1, Figure 6B). Consistent with our degradation and interaction results, SIVdeb Vpr is able to polyubiquitylate both ΔC and ΔN SAMHD1, although both were reduced relative to WT SAMHD1. Interestingly, SIVdeb Vpr is still able to polyubiquitylate ΔN/ΔC SAMHD1, though less efficiently than ΔC or ΔN SAMHD1. This is in contrast to SIVmac which cannot catalyze the polyubiquitylation of ΔC or ΔN/ΔC SAMHD1, and SIVrcm Vpx which cannot catalyze the polyubiquitylation of ΔN or ΔN/ΔC SAMHD1 (Figure 6B). These data suggest that in addition to contributions of both the N- and the C-terminus, SIVdeb Vpr could have evolved to further recognize the central portion of SAMHD1 for binding and degradation. Together, these data argue that the Vpx/Vpr-SAMHD1 interface is a dynamic interface whose requirements for binding and degradation have toggled back and forth through the evolution of this virus-host arms-race (summarized in Figure 6C).

## Discussion

We show that distinct Vpx/Vpr proteins require disparate termini of SAMHD1 in order to antagonize this host restriction factor. These differences correlate with the phylogenetic separation of Vpx, with the SIVsmm/SIVmac/HIV-2 Vpx proteins requiring the C-terminus of SAMHD1, while the SIVmnd2/SIVrcm Vpx proteins require the N-terminus of SAMHD1 for binding, ubiquitylation, and subsequent degradation. Thus, despite a conserved mechanism of degradation, Vpx from distinct lineages of SIV show both phylogenetic and functional disparity. Most surprisingly, the specific requirements in SAMHD1 for degradation/binding have toggled back and forth during Vpx/Vpr evolution in primate lentiviruses. These results demonstrate that the antagonism between Vpx/Vpr and SAMHD1 is evolutionarily dynamic, with virus and host sides evolving to counteract each other through an evolvable interface.

### A model to explain evolutionary toggling of the Vpx/Vpr interaction with SAMHD1

**Figure 7:**
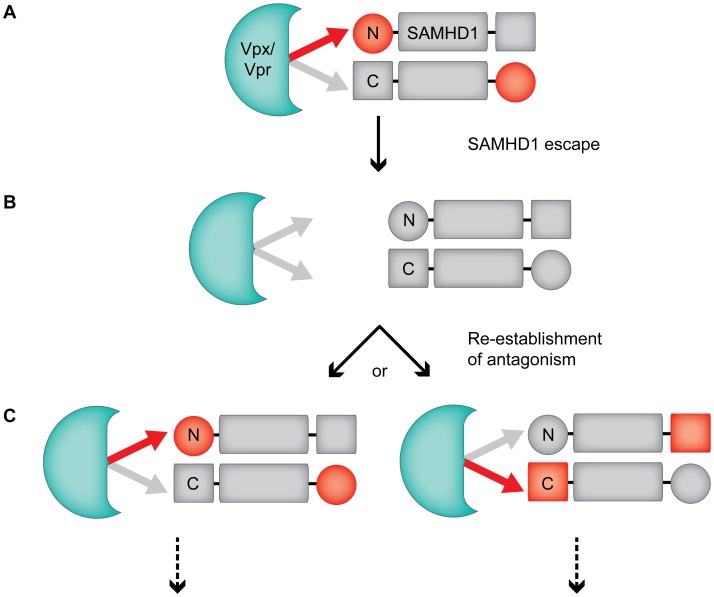
Model of Vpx/Vpr toggling specificity for SAMHD1 during the evolutionary arms-race of this virus-host interaction. (A) An N-terminal binding Vpx shows strong affinity for the N-terminus of SAMHD1, and weak affinity for the C-terminus of another molecule present in the SAMHD1 tetramer (red and grey arrows, respectively, tetramer is represented as a dimer for simplicity). Vpx/Vpr is represented by a green shape in all panels. (B) Evolutionary pressure by Vpx alters SAMHD1 sequence at the binding interface, resulting in weakened affinity of Vpx for both the N- and the C-terminus. This leads to subsequent SAMHD1 escape. (C) A variant of Vpx re-establishes interaction with SAMHD1 either through high affinity for the N- or the C-terminus, and with weak affinity for the opposite terminus of SAMHD1 in this tetramer. This stabilizes the interaction and leads to degradation of SAMHD1. Downward dotted arrows represent future antagonism and evolution. Together, the differential affinity of Vpx for disparate termini in SAMHD1 results in the evolutionary toggling of Vpx/Vpr specificity observed for SAMHD1. This model does not imply a temporal order of N- or C-terminal binding.

The arms-race between antiviral host proteins and viral antagonists often depends on sequence variation at a single interface between virus and host proteins that results in a rapid evolution at the point of contact [24], [31]. Our results with Vpx/Vpr and SAMHD1 indicate that more complex evolutionary scenarios can exist in which the mode of recognition between the viral antagonist and host protein can change. While it is possible that recognition of the N- or the C-terminus of SAMHD1 by Vpx/Vpr arose independent of each other, an attractive alternative is that Vpx/Vpr can sample both the N- and the C-terminus of SAMHD1 for optimal binding. This hypothesis is supported by biochemical studies of SAMHD1, which show that these termini of SAMHD1 are likely proximal in three-dimensional space. SAMHD1 was recently crystalized as a head-to-tail dimer [2], and further biochemical evidence indicates that tetramerization of SAMHD1 is necessary for the phosphohydrolase activity of the protein [29]. Thus the proximity of the SAMHD1 N- and C-terminus could allow Vpx/Vpr to shift its binding from one terminus to the other (Figure 7). In this model we suggest that both N- and C-terminal domains in SAMHD1 contribute to overall binding, but that one or the other shows stronger affinity depending on which Vpx/Vpr protein is binding (Figure 7A). Evolutionary pressure to evade this antagonism would create escape mutations in SAMHD1 that would destabilize the interaction with Vpx/Vpr (Figure 7B). However, new Vpx/Vpr variants that re-establish the interaction with SAMHD1 could do so by strengthening the interaction at either the N-terminus or the C-terminus of SAMHD1 (Figure 7C). Thus giving rise to the evolutionary toggling of Vpx/Vpr specificity for SAMHD1.

The model proposed in Figure 7 would hypothesize that some Vpx/Vpr proteins are dependent on both N- and C-terminal sequences for binding and degradation. Indeed, our data on SIVdeb Vpr further supports this hypothesis, as SIVdeb Vpr degradation of SAMHD1 is unaffected by truncations at the C-terminus and occurs independent of variation in the N-terminus. Furthermore, SIVdeb Vpr can polyubiquitylate both N- and C-terminal truncations of SAMHD1, though to a lesser extent then WT (Figure 6). Together, the identification of Vpr proteins that show requirements for multiple domains in SAMHD1 additionally supports our hypothesis that different affinities to either the N- or the C-terminus are what drive the toggling of Vpx/Vpr specificity for SAMHD1.

### Differences and similarities with other lentivirus-host arms-races

This evolutionary interaction of Vpx/Vpr with SAMHD1 differs from previously described arms-races between virus and host factors, where the escape and re-establishment of interaction takes place at a discrete interface [31]. For example, the PRYSPRY domain of the antiviral factorTRIM5α is highly variable between primate species [26], and this variation governs the functional recognition of retroviral capsid [32]. Moreover, variation in a single region of the restriction factor APOBEC3G changes specificity for the interaction with the lentiviral Vif protein [33]. When lentiviruses have adapted to changes in the host through alternate binding modes, amino acid deletions (and theoretically alterations in protein structure) were the driving force behind these adaptations. For example, Vif has shifted its recognition of APOBEC3G by three to 15 amino acids in a specific subset of lentiviruses infecting the *Colobinae* subfamily of Old World Monkeys, presumably due to a deletion in APOBEC3G upstream of the binding site [34]. Additionally, while Nef recognizes the restriction factor BST-2/tetherin in most primates, the Nef binding site in BST-2/tetherin is missing in humans [35]. Instead of merely shifting the region of BST-2/tetherin that Nef binds, HIV-1 adapted a different protein, Vpu, to target and degrade BST-2/tetherin. In contrast, the results shown here illustrate the extent to which a novel interface can evolve in the absence of gross changes in the sequence of the host protein under attack.

### Evolution of Vpx/Vpr and SAMHD1

Changes in Vpx also accompany changes in SAMHD1 recognition. We found that SIVrcm Vpx does not require the same amino acids to target SAMHD1 as HIV-2/SIVmac Vpx. However, this is not specific to N-terminal binding Vpx proteins, as SIVmnd2 Vpx utilizes the same residues as HIV-2/SIVmac Vpx. How SIVmnd2 Vpx is able to bind to the N-terminus of SAMHD1 while using the same residues as C-terminally binding Vpx proteins will need to be determined. Furthermore, whether SIVmnd2 Vpx or SIVrcm Vpx is the outlier within N-terminally binding Vpx proteins is still unclear, as more N-terminal binding Vpx and Vpr proteins will need to be analyzed for their binding to SAMHD1.

Our results further help to explain the evolutionary signatures observed in SAMHD1. We had previously reported that most of the residues in SAMHD1 that show strong signatures of positive selection in Old World Monkeys were located in the N-terminal SAM domain, and through altering the amino acids evolving under positive selection, we functionally showed the SAM domain as an important interface in this virus-host antagonism [21]. Another group, analyzing a wider range of primate species, observed several sites in the C-terminus of SAMHD1 evolving under positive selection, and this region was further shown to be necessary for degradation by Vpx [10], [27], [28]. Taken together, these results argue that SAMHD1 has faced selective pressure at both the N and C-terminus, with possibly a more recent selective pressure on the N-terminus. Our results in this study indicate that evolution at both of these surfaces could have been driven by lentiviral Vpx/Vpr, highlighting the strong selective pressure that lentiviruses impose on primate evolution. Moreover, our results suggest that adaptive changes in SAMHD1 have been partially responsible for driving the changes in Vpx/Vpr that permit toggling of the region of SAMHD1 that is recognized. Together, our data not only fortifies the importance of the Vpx/Vpr-SAMHD1 interface in the evolutionary history of lentiviral interactions with Old World monkeys, but it further highlights the evolutionary plasticity of an interface between restriction factors and their lentiviral antagonists.

## Methods

### Plasmids

SAMHD1, Vpx, and Vpr primary sequences, as well as LPCX-HA SAMHD1 and pCDNA-3xFLAG Vpx or Vpr constructs were described previously [21]. SIVsyk Vpr was cloned from the proviral plasmid [36]. SAMHD1 ΔC, ΔN, and chimeric constructs were generated by PCR and subcloned into LPCX or pCG vector [8], using standard cloning techniques. Vpx mutants were generated using Quickchange site-directed mutagenesis PCR (Stratagene). The following genes were codon-optimized and synthesized (Genscript): HIV-2 Vpx n019015, n012055, n012002, n004008, n012034, and SIVsm SL2 Vpx.

### Transfection

Transfection in 293T cells was performed as described [21] with minor modifications. Briefly, cells were transfected with 200 ng of LPCX-HA-SAMHD1 with or without 100 ng of Vpx/Vpr constructs using TransIT-LT1 (Mirus Bio). Codon optimized Vpx/Vpr was titrated in order to normalize for similar levels of protein expression. The total amount of DNA in all transfections was maintained constant with appropriate empty vectors. Thirty-six hours post-transfection, cells were harvested for Western blot analysis.

### Immunoprecipitations and Western blotting

Immunoprecipitations were performed as described previously [8], [10]. For whole cell lysates, 293T cells were lysed in RIPA buffer for 10 minutes on ice, and spun at 15,000 g for 10 minutes to pellet. Lysates were run on NuPAGE Novex 4–12% Bis-Tris gradient gels (Invitrogen). The following antibodies were used: HA-specific antibody (Babco), anti-FLAG M2 antibody (Sigma-Aldrich), anti-tubulin (Sigma-Aldrich), anti-DDB1 (Invitrogen); DCAF1 was detected with rabbit antibody raised to recombinant protein [37]. Primary antibodies were detected with a corresponding horseradish peroxidase-conjugated secondary antibody (Santa Cruz Biotech).

### Phylogenetic analysis

Phylogenetic trees were constructed from amino acid alignments of Vpx sequences obtained from the Los Alamos HIV sequence database [38]. Alignments were performed using fast statistical alignment (FSA) [39] or Muscle [40]. Phylogenies were constructed with PhyML [41] by the maximum-likelihood (ML) method. Support for ML trees was assessed by aLRT [42]. Analyses were performed at least three times.

### In vitro SAMHD1 recruitment assays

Recombinant Rhesus SAMHD1 including full-length (WT), 1–595 (SAMHD1-ΔC) and 113–625 (ΔN-SAMHD1) and human DDB1-DCAF1c (1045–1396), and DDB1-DCAF1c in complex with viral accessory proteins, including Vpx (SIVmac) and Vpx (RCM) were expressed and purified as described previously [10]. Typically, 100 µL of protein mixtures as indicated were injected into an analytical size exclusion column (Superdex200, 10×250 mm, 24 mL) equilibrated with a buffer containing 25 mM sodium phosphate, pH 7.5, 150 mM NaCl, 5% glycerol, and 0.02% azide at a flow rate of 0.8 mL/min. The peak fractions (0.5 mL) were concentrated to 20-fold and analyzed by SDS-PAGE. The gel was first developed with Coomassie Blue stain, and subsequently Silver stained.

### In vitro ubiquitylation assays

In vitro ubiquitylation was performed as previously described [10]. Briefly, E1 (UBA1, 0.2 µM), E2 (UbcH5b, 2.5 µM), and E3 complexes (mixtures of DDB1-DCAF1c-Vpx and CUL4A-RBX1 at 0.3 µM, indicated as CRL4-DCAF1-Vpx) were typically incubated with 0.6 µM of T7-tagged SAMHD1 and 2.5 µM of His_6_-FLAG-tagged ubiquitin in a buffer containing 10 mM Tris-HCl, pH 7.5, 150 mM NaCl, 5% Glycerol, 20 U/mL pyrophosphatase, 2 mM DTT and 5 mM ATP at 37°C for 0, 15 and 30 min. The reaction was quenched with non-reducing SDS-PAGE gel loading buffer and analyzed by Western blotting.

## Supporting Information

Figure S1 **Phylogenetic analysis of HIV-2 and SIVsm Vpx proteins, related to** **Figure 1** **.** (A) Alignment of HIV-2 and SIVsmm Vpx protein sequences was performed with FSA, and the maximum likelihood tree was generated with PhyML. aLRT values greater than 0.75 are shown at key nodes and denoted with a *. HIV-2 and SIVsmm sample names are shown and the Vpx proteins tested in (B) are highlighted in bold. (B) 293T cells were transfected with human HA-SAMHD1, either WT or ΔC, plus or minus FLAG-Vpx from HIV-2 or SIVsmm, and degradation was measured by western blotting.
(PDF)

Figure S2 **Amino acid diversity of SAMHD1 in the N- and C-terminus, related to** **Figure 2** **.** Alignments of N-termini and C-termini of SAMHD1 proteins analyzed in this study. SAMHD1 protein alignments were performed with Muscle [40]. Dashed line indicates conserved amino acid.
(PDF)

Figure S3 **N- and C-terminal binding Vpx proteins recruit SAMHD1 to the DDB1-DCAF1 complex through a conserved mechanism, related to** **Figure 4** **.** *In vitro* rhesus SAMHD1 recruitment assays with SIVmac or SIVrcm Vpx. (A) 100 µL of SAMHD1-FL (a), SAMHD1-ΔC (b), and ΔN-SAMHD1 (c) at 1.5 µM were injected into an analytical Superdex 200 size exclusion column (10×250 mm, 24 mL), equilibrated with a buffer containing a 25 mM sodium phosphate, pH 7.5, 150 mM NaCl, 5% glycerol, and 0.02% azide at a flow rate of 0.8 mL/minA. Trace of UV280 nm with elution volume of the peak is shown. Chromatograms are shown at top, labeled a–c, and fractions analyzed by Coomassie stain, shown below, are numbered (1–3, corresponding to the peak elution). (B) DDB1-DCAF1c in complex with SIVmac Vpx (a, 1), without Vpx (b, 2), or with SIVrcm Vpx (c, 3) at a concentration of 3.5 µM were analyzed as described in A. Silver staining of the lower molecular weight region of SDS-PAGE gel is shown for visualization of Vpx. (C) Mixtures of DDB1-DCAF1c (3.5 µM) with FL (a, 1–4), ΔC (b, 5–7), and ΔN (c, 8–10) SAMHD1 at 1.5 µM were analyzed as described in A. (D) Mixtures of DDB1-DCAF1c-SIVmac Vpx (3.5 µM) with FL (a, 1–7), ΔC (b, 8–10), and ΔN (c, 11–15) SAMHD1 (1.5 µM) were analyzed as in (A); (E) DDB1-DCAF1c-SIVrcm Vpx (3.5 µM) with FL (a, 1–7), ΔC (b, 8–15), and ΔN (c, 16–17) SAMHD1(1.5 µM) were also analyzed as in (A).
(PDF)

Figure S4 **Mutations in SIVrcm Vpx that do not abolish degradation of SAMHD1 do not change breadth or requirement for SAMHD1 N- or C-terminus, related to** **Figure 5** **.** Mutant SIVrcm _12 HIV-2 17_ Vpx was tested for the gain of function, loss of function, or altered dependence on the N- or C-terminus to degrade SAMHD1. 293T cells were co-transfected with (+) or without (−) FLAG-Vpx and HA-tagged Human-RCM SAMHD1 chimeras (Hu-RCM) for gain of function, RCM-Human SAMHD1 chimeras (RCM-Hu) for loss of function, or C-terminally truncated SAMHD1 chimeras (Hu-RCM ΔC and RCM-Hu ΔC) for altered dependence on the N- or C-terminus, and analyzed by western blotting.
(PDF)

Figure S5 **SIVagm.gri Vpr shows a dependence on both the N- and the C-terminus of SAMHD1.** HA-tagged AGM SAMHD1 (WT, ΔC, and ΔN) were transiently co-expressed in 293T cells with FLAG-SIVagm.gri Vpr (Vpr from SIV that infects the AGM grivet subspecies) and immunoprecipitated from whole cell extracts with anti-HA resin. HA-SAMHD1, FLAG-Vpr, DCAF, and DDB1 were detected in immune complexes (upper panels) or extracts (lower panels) by western blotting.
(PDF)
