# Supplementary figures and images for: Evolutionary Toggling of Vpx/Vpr Specificity Results in Divergent Recognition of the Restriction Factor SAMHD1
Source: PLoS Pathog. 2013 Jul 18;9(7):e1003496. doi: 10.1371/journal.ppat.1003496 (PMC3715410; doi:10.1371/journal.ppat.1003496)

Figure S1

A

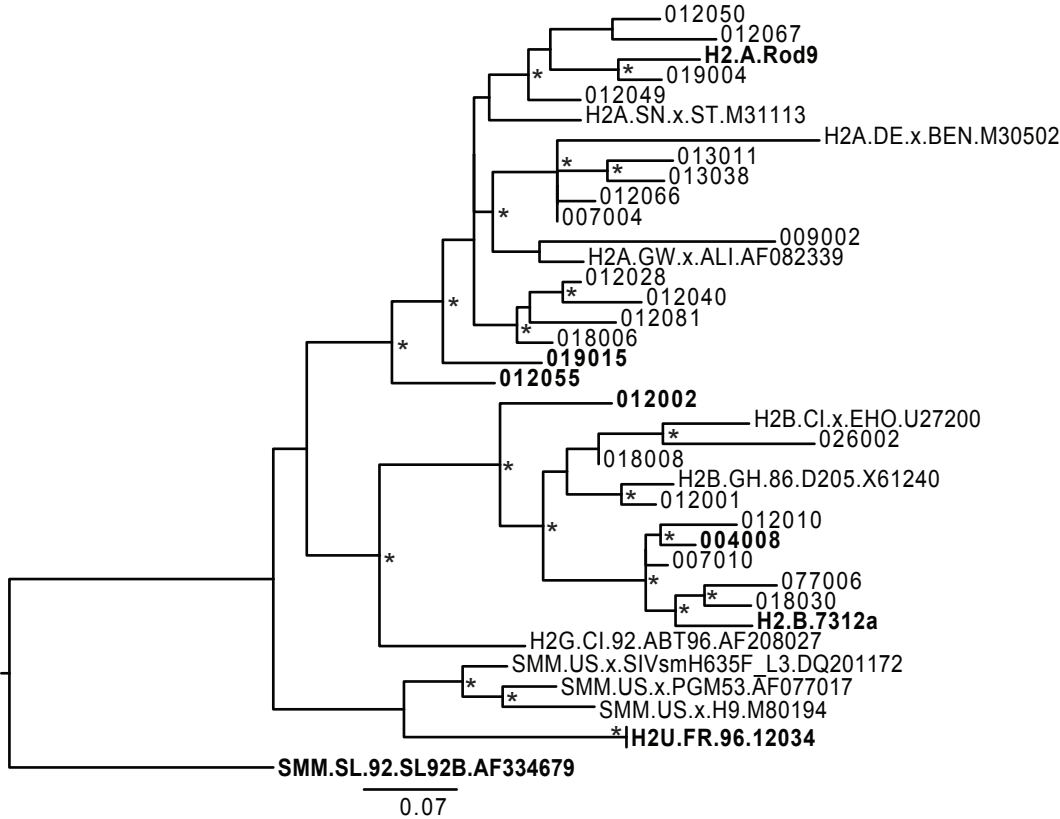

B

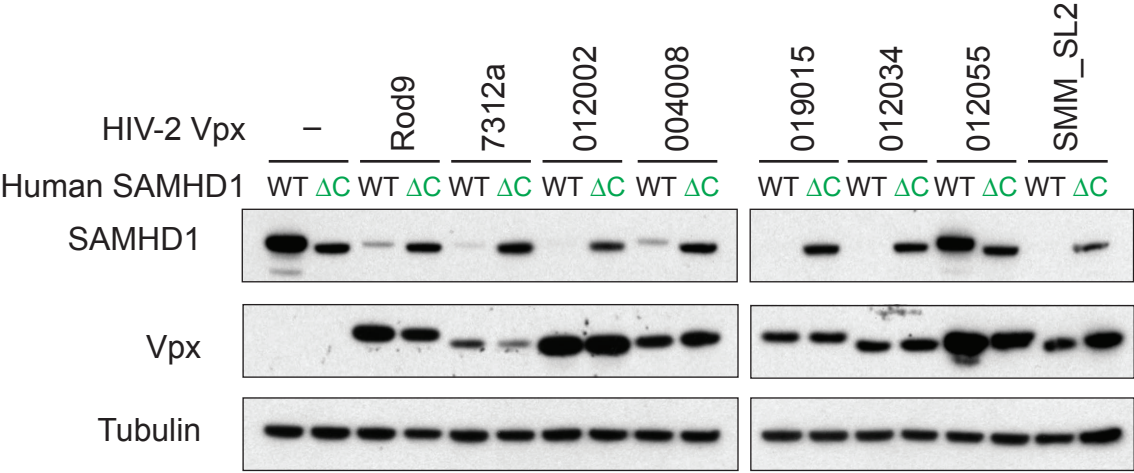

Supplement: Figure S1 — Phylogenetic analysis of HIV-2 and SIVsm Vpx proteins, related to Figure 1 . (A) Alignment of HIV-2 and SIVsmm Vpx protein sequences was performed with FSA, and the maximum likelihood tree was generated with PhyML. aLRT values greater than 0.75 are shown at key nodes and denoted with a *. HIV-2 and SIVsmm sample names are shown and the Vpx proteins tested in (B) are highlighted in bold. (B) 293T cells were transfected with human HA-SAMHD1, either WT or ΔC, plus or minus FLAG-Vpx from HIV-2 or SIVsmm, and degradation was measured by western blotting. (PDF) [file ppat.1003496.s001.pdf]

Figure S3

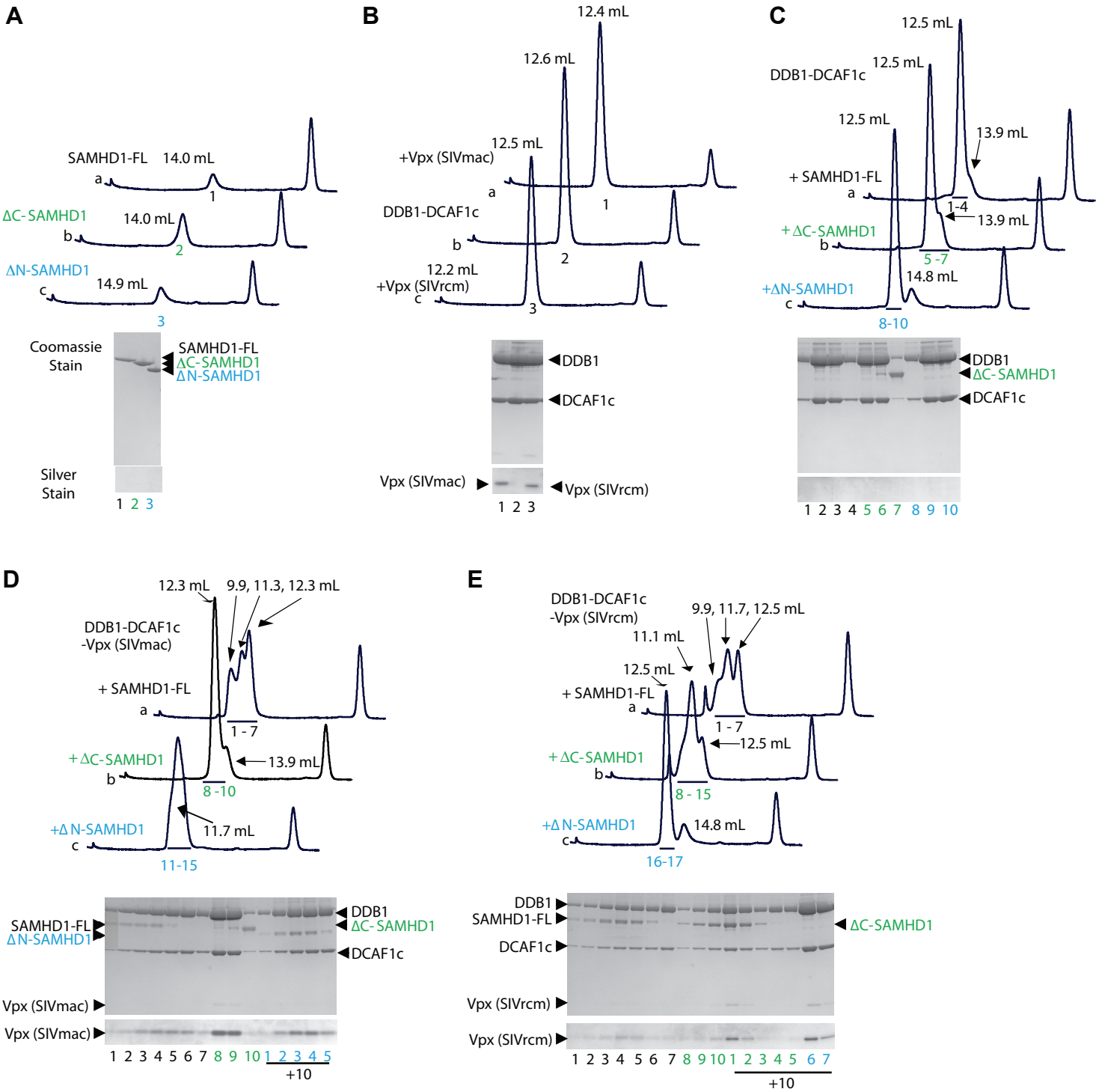

Supplement: Figure S3 — N- and C-terminal binding Vpx proteins recruit SAMHD1 to the DDB1-DCAF1 complex through a conserved mechanism, related to Figure 4 . In vitro rhesus SAMHD1 recruitment assays with SIVmac or SIVrcm Vpx. (A) 100 µL of SAMHD1-FL (a), SAMHD1-ΔC (b), and ΔN-SAMHD1 (c) at 1.5 µM were injected into an analytical Superdex 200 size exclusion column (10×250 mm, 24 mL), equilibrated with a buffer containing a 25 mM sodium phosphate, pH 7.5, 150 mM NaCl, 5% glycerol, and 0.02% azide at a flow rate of 0.8 mL/minA. Trace of UV280 nm with elution volume of the peak is shown. Chromatograms are shown at top, labeled a–c, and fractions analyzed by Coomassie stain, shown below, are numbered (1–3, corresponding to the peak elution). (B) DDB1-DCAF1c in complex with SIVmac Vpx (a, 1), without Vpx (b, 2), or with SIVrcm Vpx (c, 3) at a concentration of 3.5 µM were analyzed as described in A. Silver staining of the lower molecular weight region of SDS-PAGE gel is shown for visualization of Vpx. (C) Mixtures of DDB1-DCAF1c (3.5 µM) with FL (a, 1–4), ΔC (b, 5–7), and ΔN (c, 8–10) SAMHD1 at 1.5 µM were analyzed as described in A. (D) Mixtures of DDB1-DCAF1c-SIVmac Vpx (3.5 µM) with FL (a, 1–7), ΔC (b, 8–10), and ΔN (c, 11–15) SAMHD1 (1.5 µM) were analyzed as in (A); (E) DDB1-DCAF1c-SIVrcm Vpx (3.5 µM) with FL (a, 1–7), ΔC (b, 8–15), and ΔN (c, 16–17) SAMHD1(1.5 µM) were also analyzed as in (A). (PDF) [file ppat.1003496.s003.pdf]

Figure S4

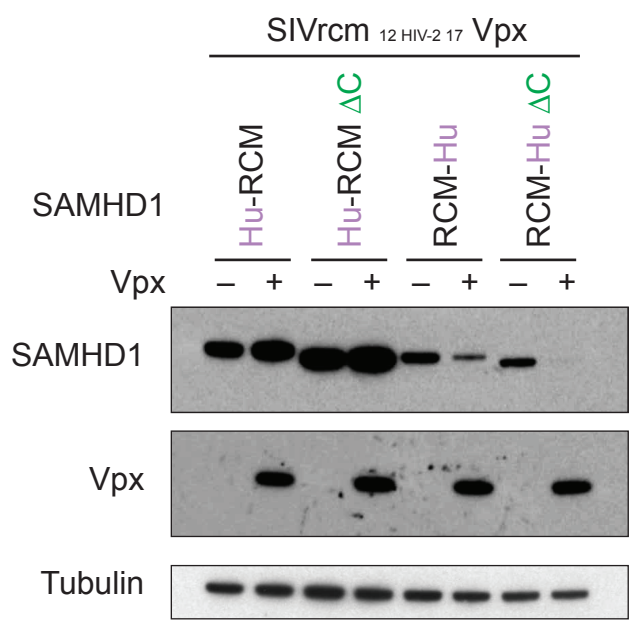

Supplement: Figure S4 — Mutations in SIVrcm Vpx that do not abolish degradation of SAMHD1 do not change breadth or requirement for SAMHD1 N- or C-terminus, related to Figure 5 . Mutant SIVrcm 12 HIV-2 17 Vpx was tested for the gain of function, loss of function, or altered dependence on the N- or C-terminus to degrade SAMHD1. 293T cells were co-transfected with (+) or without (−) FLAG-Vpx and HA-tagged Human-RCM SAMHD1 chimeras (Hu-RCM) for gain of function, RCM-Human SAMHD1 chimeras (RCM-Hu) for loss of function, or C-terminally truncated SAMHD1 chimeras (Hu-RCM ΔC and RCM-Hu ΔC) for altered dependence on the N- or C-terminus, and analyzed by western blotting. (PDF) [file ppat.1003496.s004.pdf]

Figure S5

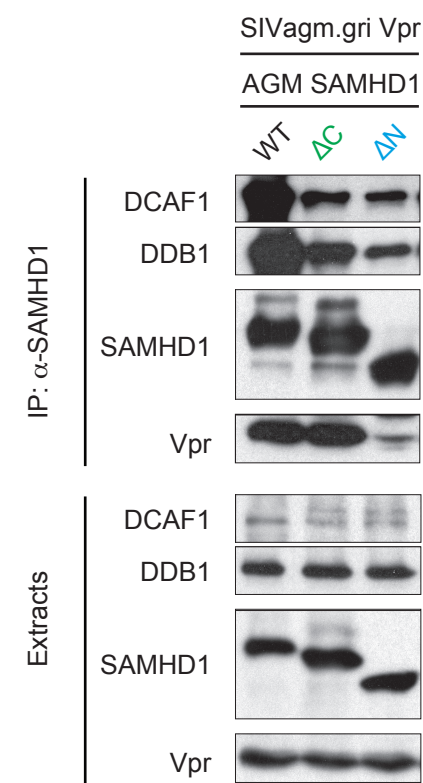

Supplement: Figure S5 — SIVagm.gri Vpr shows a dependence on both the N- and the C-terminus of SAMHD1. HA-tagged AGM SAMHD1 (WT, ΔC, and ΔN) were transiently co-expressed in 293T cells with FLAG-SIVagm.gri Vpr (Vpr from SIV that infects the AGM grivet subspecies) and immunoprecipitated from whole cell extracts with anti-HA resin. HA-SAMHD1, FLAG-Vpr, DCAF, and DDB1 were detected in immune complexes (upper panels) or extracts (lower panels) by western blotting. (PDF) [file ppat.1003496.s005.pdf]
