# Supplementary material for: Evolutionary Toggling of Vpx/Vpr Specificity Results in Divergent Recognition of the Restriction Factor SAMHD1
Source: PLoS Pathog. 2013 Jul 18;9(7):e1003496. doi: 10.1371/journal.ppat.1003496 (PMC3715410; doi:10.1371/journal.ppat.1003496)
Supplement: Figure S2 — Amino acid diversity of SAMHD1 in the N- and C-terminus, related to Figure 2 . Alignments of N-termini and C-termini of SAMHD1 proteins analyzed in this study. SAMHD1 protein alignments were performed with Muscle [40]. Dashed line indicates conserved amino acid. (PDF) [file ppat.1003496.s002.pdf]

Figure S2

N-terminus

```
Hu SAMHD1    MQRADSEQPS KRPRCDDSPR TPSNTPSAEA DWSPGLELHP DYKTWGPEQV CSFLRRGGFE      60
Rhe SAMHD1   --Q---D--- ----F----- ---------- -C-------- ---------- -F-------G
Mnd SAMHD1   --Q---D--- ----F----- ---S------ -C---V---- ---------- -F-------G
RCM SAMHD1   --Q---D--- ----F----- ---S------ -C-------- ---------- -F-------G
AGM SAMHD1   --Q---D--- --L-F----- ---S------ ---------- -----D---- -F-------G
Deb SAMHD1   --Q---D--- ----F----- ---S------ -G-------- -----D---- -F-------G

Hu SAMHD1    EPVLLKNIRE NEITGALLPC LDESRFENLG VSSLGERKKL LSYIQRLVQI HVDTMKVIND      120
Rhe SAMHD1   --A------- -K-------- ----H----- ---------- ------SG-- ----------
Mnd SAMHD1   --A------- -K-------- ----H----- ---------- ------SG-- ----------
RCM SAMHD1   --A------- -K--D----- ----H----- ---------- ------SG-- ----------
AGM SAMHD1   --A-----Q- -K-------- ----H----- ---------- -------G-- ----------
Deb SAMHD1   --A------- -K-------- ----H----- ---------- -------G-- ----------
```

C-terminus

```
Hu SAMHD1    ITPQKKEWND STSVQNPTRL REASKSRVQL FKDDPM    626
Rhe SAMHD1   ---------Y R----S---- -------L-- -----K
Mnd SAMHD1   ---------Y R----S---- -------L-- -----K
RCM SAMHD1   ---------Y R----S---- -------L-- -----K
AGM SAMHD1   ---------Y R----S---- -------L-- -----K
Deb SAMHD1   ---------Y R----S---- -------L-- -----K
```
